# Correction: Reduced activity, reactivity and functionality of the sympathetic nervous system in fibromyalgia: An electrodermal study

**DOI:** 10.1371/journal.pone.0244830

**Published:** 2020-12-28

**Authors:** Gustavo A. Reyes del Paso, Pablo de la Coba

In Table 1, the values in the last three rows of column “*p*” are mistakenly duplicated in column “*η2*.” Please see the correct Table 1 here.

**Table 1:** Demographic, clinical, medication use (number of participants and percentage in brackets) and physiological data in fibromyalgia (FMS) patients and healthy participants.

	FMS (n = 45)	Healthy (n = 38)	t or *χ²*	*p*	*η2*
Age (years)	51.53+8.89	50.08+10.22	.693	.490	.006
BMI	27.26+4.76	26.17+3.64	1.184	.240	.016
State Anxiety (STAI)	21.87±12.16	13.00±7.47	4.07	< .001	.159
Nº of pain points (MPQ)	21.98±6.76	4.00±3.84	15.17	< .001	.722
Sensorial pain (MPQ)	32.11±21.73	13.00±7.33	5.54	< .001	.248
Total MPQ Score	49.29±30.67	20.45±11.35	5.85	< .001	.271
Current pain intensity (MPQ)	2.62±1.27	0.84±0.79	7.81	< .001	.411
Fatigue (FSS)	49.42±11.31	20.66±13.48	10.57	< .001	.580
Antidepressant use (%)	23 (51.11)	3 (6.67)	17.89	< .001	.215
Anxiolytic use (%)	29 (64.44)	7 (15.56)	17.768	< .001	.214
Analgesic use (%)	36 (90)	4 (8.89)	39.828	< .001	.480
Opiate use (%)	19 (42.22)	0 (0)	20.808	< .001	.251
SC level (µS)	2.02±2.11	3.56 ±2,11	-2.15	.034	.054
Slope SC (µS/m)	-0.153±.237	-0.344±.584	2.01	.048	.047
Hand Temperature (C^o^)	35.91±1.03	35.92± .82	-.039	.969	< .001

**Note:** Results of group comparisons are also reported (Student *t* test or *χ²*). Effect size was reported as adjusted eta squared (***η2***). MPQ = McGill Pain Questionnaire; FSS = Fatigue Severity Scale.
